# Supplementary material for: Overcoming thermal noise in non-volatile spin wave logic
Source: Sci Rep. 2017 May 15;7:1915. doi: 10.1038/s41598-017-01995-8 (PMC5432494; doi:10.1038/s41598-017-01995-8)
Supplement: Supplementary file 1 — Supplementary information [file 41598_2017_1995_MOESM1_ESM.pdf]

Overcoming thermal noise in non-volatile spin wave logic

# – Supplementary information

Sourav Dutta*[1], Dmitri E. Nikonov[2], Sasikanth Manipatruni[2], Ian A. Young[2], and Azad Naeemi[1]

[1]School of Electrical and Computer Engineering, Georgia Institute of Technology, Atlanta, GA 30332 USA

[2]Components Research, Intel Corporation, Hillsboro, OR 97124 USA

Contents

S1. Comparison with alternative spin configuration of ME-SWB system
S2. Calculation of perpendicular magnetic anisotropy (PMA) of Spin Wave Bus
S3. Comparison with other PMA SWB
S4. Comparison of material parameters for ME cell
S5. Ultrahigh strain and strain relaxation
S6. Mathematic expression for ME effect
S7. Device working principle
S8. Comparison with voltage-controlled magnetic anisotropy (VCMA) effect
S9. Non-volatility and magnetization tilting in the presence of built-in strain and in exchange-spring structure
S10. Asymmetric tilted distribution of switching success as a function of detected < ϕ >
S11. Comparison between 1D and full 3D micromagnetic simulation
S12. Approximate estimation of error rate

S1. Comparison with alternative spin configuration of ME-SWB system

S1.1. PMA SWB – in-plane ME cell

In this paper, we explore the system of PMA spin wave bus (SWB) and ME cell with stable magnetization states along the long axis (+/- x) as shown in Fig. 1(a). Note that the choice of mutually orthogonal spin configuration of SWB and ME cell stems from the requirements of non-volatility and non-reciprocity [1]. Applying a voltage aligns the magnetization of the ME cell with that of the SWB allowing the spin waves to arrive and subsequently get detected. This working principle can be extended to in-plane magnetized SWB.

S1.2. In-plane SWB – in-plane ME cell

Fig. 1(b) shows a magnetostatic surface SWB magnetized along the in-plane hard axis (y) and the corresponding mutually orthogonal magnetization of the ME cell. The magnetization of the ME cell would be energetically favored along the x-axis due to the inherent shape-anisotropy and the

presence of a possible magnetocrystalline anisotropy. Application of a voltage in this scenario across a (011) cut ferroelectric layer can create an anisotropic strain[2] to rotate the easy axis from x to y and switch the magnetization. Note that since the ME cell would have a naturally occurring saddle point along the y axis in the absence of any built-in strain, the saddle point based phase detection of the spin waves would be valid in the scenario. However, a major drawback is the requirement of an external biasing field to create the transverse magnetization in SWB nanowire.

S1.3. In-plane SWB – PMA ME cell

Fig. 1(c) shows a longitudinally magnetized backward volume SWB along with a PMA ME cell. The PMA ME cell can be obtained by using the surface anisotropy at the interface of a magnetostrictive layer and a metal/ oxide (Ni/Cu [3], CoFe/MgO [4] etc.) or large in-plane built-in strain like Ni/$BaTiO_3$ [5]. An applied voltage can create an in-plane isotropic or anisotropic strain to lower the PMA and cause in-plane switching of magnetization towards the x-axis (favored due to shape anisotropy). Note that for a PMA magnet, the saddle point is located at the x-axis and hence the saddle point based phase detection of the spin waves would also be valid in the scenario. However, as mentioned earlier in the main text, the broken translational symmetry and anisotropic dispersion relation of the backward volume spin waves can give rise to scattering processes where the waves interfere.

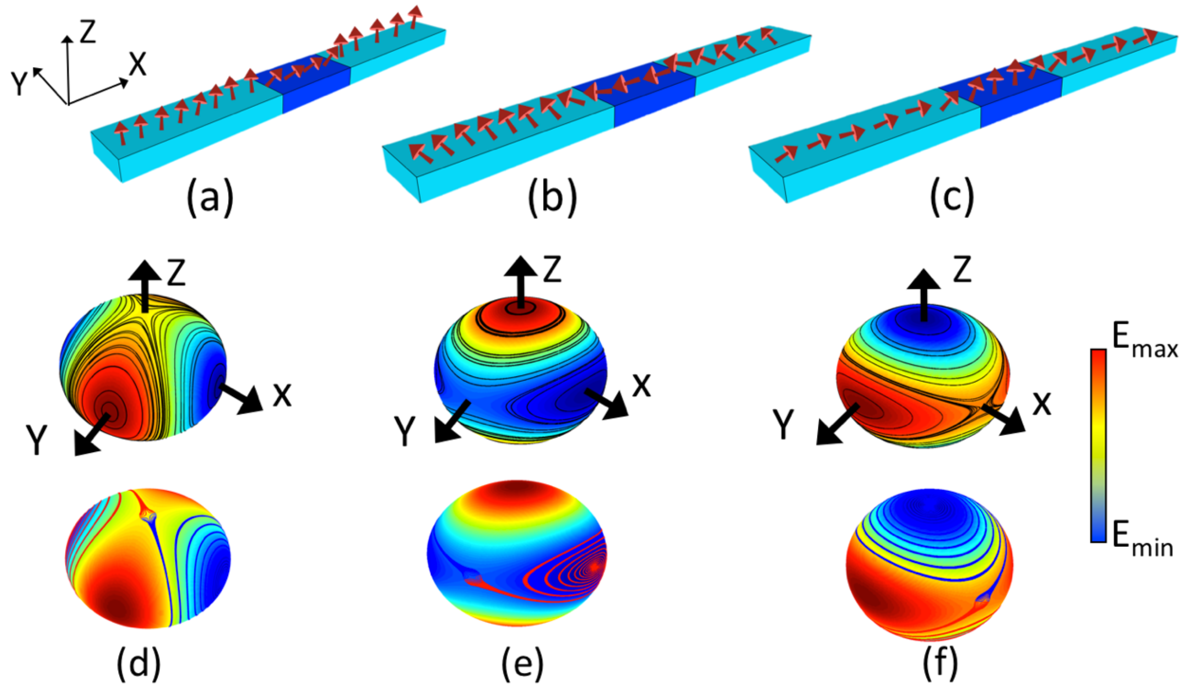

Figure 1. Illustration of the possible spin configuration of the ME-SWB system.

S2. Calculation of perpendicular magnetic anisotropy (PMA) of Spin Wave Bus

The origin of magnetic anisotropy can be attributed to two mechanisms: (a) the long range magnetic dipolar interaction which gives rise to the shape anisotropy, and (b) the spin-orbit interaction which gives rise to magnetocrystalline anisotropy and magnetoelastic anisotropy. The perpendicular magnetic anisotropy (PMA) arises from this spin-orbit interaction at the interface which has a lowered symmetry, and hence behaves differently from bulk magnetic anisotropy. Specifically, theoretical prediction by Daalderop et. al. [6] in Co/Ni multilayer system has revealed the PMA to be arising from the spin-orbit interaction of states with $d_{x^2-y^2}$ and d_{xy} character present close to the Fermi level.
Phenomenologically, the total effective magnetic anisotropy can be separated into a volume contribution K_V and a surface contribution K_S and can be expressed as a function of the Co and Ni layer thicknesses t_{Co} and t_{Ni} and number of bilayer repetitions n as[7-10]:

$$K^{eff}D = K_V^{Co}t_{Co} + K_V^{Ni}t_{Ni} + 2K_S^{Co/Ni} + \frac{1}{n}\left[K_S^{Co/Pt} + K_S^{Ni/Pt} - K_S^{Co/Ni}\right] \qquad (1)$$

where D is the bilayer thickness (D = t_{Co} + t_{Ni} = (1 + α)t_{Co}), α = t_{Ni}/t_{Co} is the thickness ratio and K_V^{Co} and K_V^{Ni} are the volume anisotropies of Co and Ni layers, respectively. $K_S^{Co/Ni}$, $K_S^{Co/Pt}$ and $K_S^{Ni/Pt}$ are the interface anisotropies of Co/Ni, bottom Co/Pt and top Ni/Cap interfaces, respectively, considering the deposition of the Co/Ni multilayer on an underlayer of Pt and capped with a top capping layer, say Ta. Neglecting the effect of top cap layer, and assuming the following parameters: K_V^{Co} = -1 MJ/m^3, K_V^{Ni} = -0.12 MJ/m^3, $K_S^{Co/Ni}$ = 0.22 mJ/m^2, $K_S^{Co/Pt}$ = 0.88 mJ/m^2, we calculate the PMA of the multilayer as a function of the Co layer thickness t_{Co} for a fixed number of bilayers n = 10 as shown in Fig. 1(a,b) and as a function of n for a fixed thickness ratio α of 2.

The effective saturation magnetization of the multilayer is calculated as

$$M_S D = M_S^{Co}t_{Co} + M_S^{Ni}t_{Ni} \qquad (2)$$

where M_S^{Co} and M_S^{Ni} are the saturation magnetization of the Co and Ni layers with assumed valued of 1.4 MA/m and 485 kA/m, respectively.

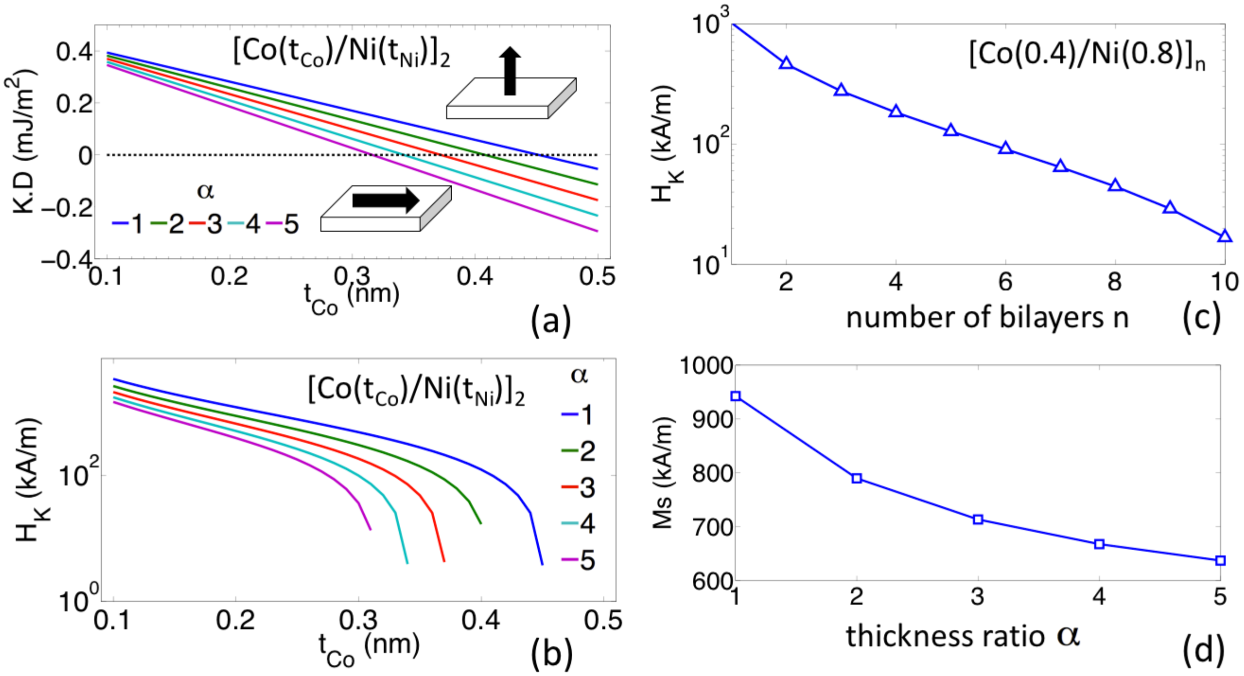

Figure 2: (a) Variation of K.D as function of the thickness of the Co layer t_{Co} in the Co/Ni multilayer stack. A positive value indicates a PMA case while negative indicates in-plane magnetization. (b), (c) Variation of the anisotropy field HK with the thickness of the Co layer t_{Co} and the number of bilayers n. (d) Variation of saturation magnetization of multilayer stack with the thickness ratio α.

S3. Comparison with other PMA SWB

The usage of surface magnetic anisotropy has been proposed to provide an out-of-plane biasing in single layer ultrathin SWB (~ 1nm) [11,12]. However, we anticipate such thin spin wave channel to be highly prone to channel noise and give rise to phase noise of propagating spin waves.

S4. Comparison of material parameters for ME cell

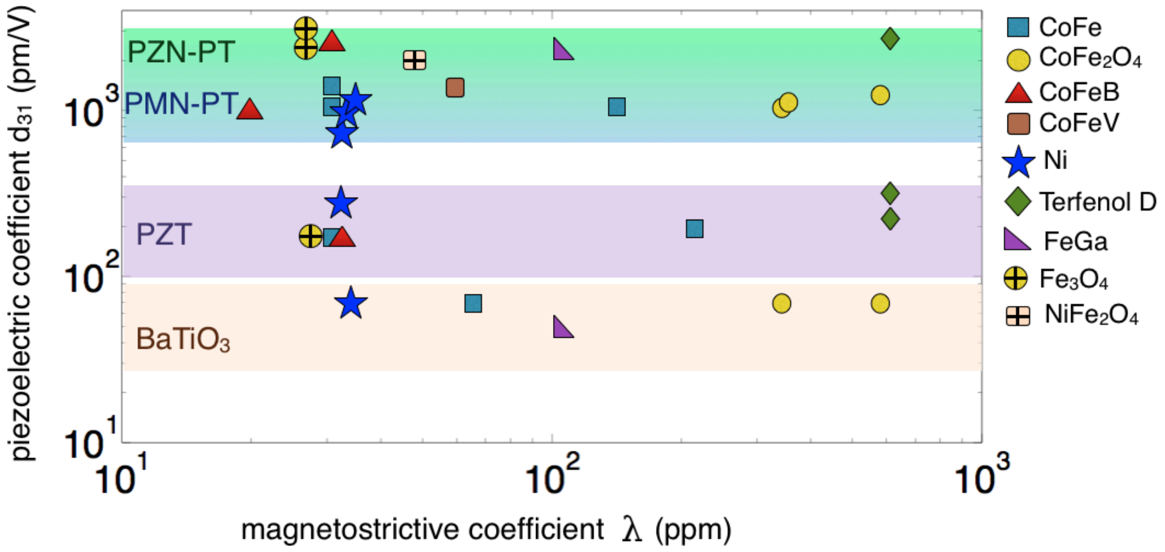

Figure 3: Map of the magnetostrictive coefficient λ of the magnetic layer and piezoelectric coefficient d_{31} of the piezoelectric/ferroelectric layer along with their compatibility for some of the demonstrated stacks.

For minimizing power dissipation, the target piezoelectric material must possess a high piezoelectric coefficient (d_{31}) while the magnetic layer must display a high magnetostrictive coefficient (λ) simultaneously. Fig. 3 shows a comprehensive map of the piezoelectric and magnetostrictive coefficients for a wide range of material including their compatibility. Nickel (Ni) [2,5,13-16] has been extensively used experimentally to demonstrate magnetoelectric effect, however it displays a low magnetostrictive coefficient (λ ~ -32 ppm). $CoFe_2O_4$ [17-23] offers an order of magnitude improved λ, however it may provide a lower thermal stability/reliability owing to low saturation magnetization. Similar high magnetostriction of λ = 150 ppm has been reported in equiatomic composition of $Co_{0.5}Fe_{0.5}$ [24,25]. Recent work by Hunter et. al. [26] has reported an enhancement of magnetostriction at the (fcc+bcc)/bcc phase boundary with effective λ as high as 260 ppm. Alternative materials include low magnetostrictive CoFeB [27-29], CoFeV [30], Fe_3O_4 [31], $NiFe_2O_4$ [32] and high magnetostrictive $Fe_{0.8}Ga_{0.2}$ [33-36](λ > 250 ppm) and highest magnetostriciton observed in $Tb_xDy_{1-x}Fe_2$ [37-39]. In this work, we consider $Co_{0.6}Fe_{0.4}$ with λ = 200 ppm. The comparison in Fig. 3 shows that, with the chosen combination of piezoelectric PMN-PT and the ferromagnet CoFe, one can reach a high product of coupling coefficient. An added advantage of our choice is a much more mature fabrication process for CoFe compared with that of Terfenol-D.

S5. Ultrahigh strain and strain relaxation

In contrast to polycrystalline materials like $Pb(Zr,Ti)O_3$ (PZT), relaxor based ferroelectric single crystals like $(Pb(Zn_{1/3}Nb_{2/3})O_3)$-$[PbTiO_3]$ (PZN-PT) and $(Pb(Mg_{1/3}Nb_{2/3})O_3)$-$[PbTiO_3]$(PMN-PT) do not require morphotropic phase boundary conditions for exhibiting ultrahigh piezoelectric strain [40]. <001> oriented relaxor based rhombohedral crystals such as (1-x)PZN-PT (x < 9 %) and (1-x)PMN-PT (x < 35 %) are known to demonstrate ultrahigh piezoelectric coefficient d_{33} and strains of 0.6 % - 0.8 % with applied electric field less than the dielectric breakdown limit [40].The reason for such high strain is presumed to be associated with an electric field induced rhombohedral-tetragonal phase transition.

Substrate induced clamping can drastically reduce the piezoelectric response of thin ferroelectric films from their bulk value [41]. As such we assume the thickness of the PMN-PT film to be at least greater than 30-50 nm. We also assume the lateral size of the PMN-PT film to be larger than the thickness to allow the formation of an in-plane isotropic biaxial strain instead of anisotropic strain [42]. It has been demonstrated that a high strain relaxation of up to 90% can occur in thick ferroelectric films (thickness > lateral dimensions) [42]. As such we limit the thickness of the CoFe layer of the ME cell to around 12 nm.

S6. Mathematic expression for ME effect

The magnetoelastic energy [43] describing the coupling between the magnetization and the strains can be written in first-order approximation as:

$$E_{ME} = -\frac{3}{2}\lambda Y\left[\left(m_x^2 - \frac{1}{3}\right)\epsilon_{xx} + \left(m_y^2 - \frac{1}{3}\right)\epsilon_{yy} + \left(m_z^2 - \frac{1}{3}\right)\epsilon_{zz}\right] \qquad (3)$$

where m_i (i=x, y, z) are the direction cosines of magnetization $\vec{M}$, λ is the magnetostrictive constant, Y is the Young's modulus and ϵ_{xx}, ϵ_{yy} and ϵ_{zz} are the strains in the x, y and z directions.

The application of an out-of-plane electric field E_Z across the (001) cut ferroelectric PMN-PT layer creates an isotropic bi-axial in-plane strain [23,44] given by

$$\epsilon_{xx} = \epsilon_{yy} = \epsilon_s = \epsilon_{res} + d_{31}E_Z \qquad (4)$$

where ϵ_{res} represents the in-plane built-in strain and d_{31} represents the piezoelectric coefficients.

The energy expression can be further reduced to [1,5]

$$E_{ME} = -\frac{3}{2}\lambda Y\left[\left(m_x^2 - \frac{1}{3}\right)\epsilon_{xx} + \left(m_y^2 - \frac{1}{3}\right)\epsilon_{yy}\right] = \frac{3}{2}\lambda Y\left(m_Z^2 - \frac{1}{3}\right)\epsilon_S \qquad (5)$$

The equivalent out-of-plane strain-induced anisotropy can be calculated as

$$K = -\frac{3}{2}\lambda Y \epsilon_S = -\frac{3}{2}\lambda Y(\epsilon_{res} + d_{31}E_Z) = -\frac{3}{2}\lambda Y\left(\epsilon_{res} + d_{31}\frac{V}{t_{PZ}}\right) \quad (6)$$

where V and t_{PZ} are the voltage applied across and the thickness of the piezoelectric layer respectively. For a stable out-of-plane magnetic configuration, K should be sufficient to overcome the out-of-plane shape anisotropy. The required voltage to create this strain-induced anisotropy is given by

$$V = -\left(\frac{2K}{3\lambda Y} + \epsilon_{res}\right)\frac{t_{PZ}}{d_{31}} \ . \quad (7)$$

S7. Device working principle

Under zero-applied voltage, the magnetization of the ME cell stays in-plane storing either a logic 1 (+x magnetization, Fig. 4(a) or logic 0 (-x magnetization, Fig. 4(b)). Applying an out-of-plane electric field across the thickness of a (001) oriented ferroelectric or piezoelectric layer (poled in the perpendicular direction and having in-plane isotropic properties) causes an in-plane biaxial strain that gets coupled to the overlaying ferromagnetic layer through the interface and thin Pt electrode. An up to 90° magnetic easy axis rotation can be achieved as the isotropic in-plane strain surpasses a critical limit causing a voltage-induced strain-mediated out-of-plane anisotropy and subsequently magnetization switching. Such in-plane to out-of-plane magnetization switching dynamics can be used to excite spin waves, with the information encoded into the phase of the waves [1]. A +x to +z magnetization switching creates a spin wave with zero phase (Fig. 4(a)), while a -x to +z switching creates spin waves with opposite π phase (Fig. 4(b)). While the transmitter ME cell switches to excite spin waves, the detector ME cell is held in the out-of-plane meta-stable state via application of voltage until the incoming spin waves arrive. Upon arrival, the voltage is switched off causing a phase-dependent out-of-plane to in-plane magnetization switching. Interestingly, depending upon the time the voltage is switched off (time of clocking), we end up with the detector ME cell's magnetization falling either in the +x or -x direction. In other words, we can define the logic function of the SW device (buffer or inverter) simply by choosing the appropriate time of clocking.

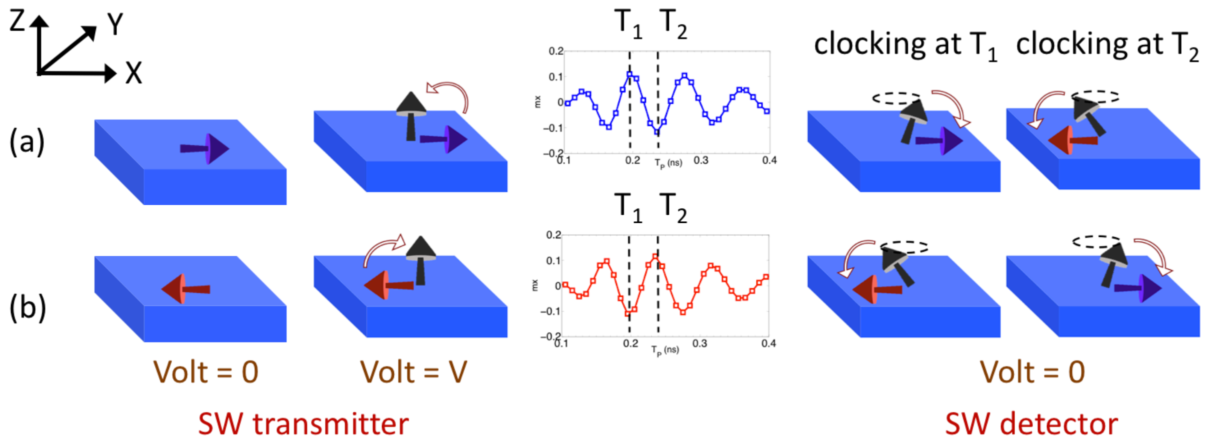

Figure 4: Working principle of the spin wave logic device.

S8. Comparison with voltage-controlled magnetic anisotropy (VCMA) effect

An applied voltage across the interface of an oxide and ferromagnet, typically MgO and CoFeB, can vary the charge density at the interface giving rise to an alteration in the surface magnetic anisotropy. Similar to magnetostriction, VCMA can give rise to a change in the magnetic easy axis with upto 90° switching of magnetization. However, with the currently known materials (Fe/MgO [45], CoFe/MgO [4] and CoFeB/MgO [46-48]), surface anisotropy displays orders of magnitude lower magnetoelectric coefficient compared to magnetostriction [49]. Hence, while the device operation can still be performed with VMCA, the energy dissipation would be considerably higher.

S9. Non-volatility and magnetization tilting in the presence of built-in strain and in exchange-spring structure

The presence of a built-in strain (less than the critical value) gives rise to a small perpendicular anisotropy $K = \frac{3}{2}\lambda Y \epsilon_{res}$ less than the shape anisotropy. The competition between the shape anisotropy favoring in-plane magnetization and PMA favoring out-of-plane configuration gives rise to a tilting of the magnetization from its stable in-plane configuration under zero-applied voltage as shown in Fig. 5(a). Beyond the critical strain of -0.48%, the magnetization goes out-of-plane thus losing the non-volatility of the zero-voltage magnetization states. The drop in the energy barrier as a function of the applied in-plane strain is also shown.

A more gradual change in the magnetization tilt angle as a function of the thickness of the CoFe layer is seen in the case of the exchange-spring system owing to the strong interlayer exchange coupling between the in-plane magnetized CoFe and PMA [Co/Ni] multilayer (Fig. 5(b)). Note that the energy barrier between the zero- voltage magnetization states drop to below 40k_BT for thickness less than 9 nm which explains the drop in the switching success of thinner ME cell magnet highlighted in the main text.

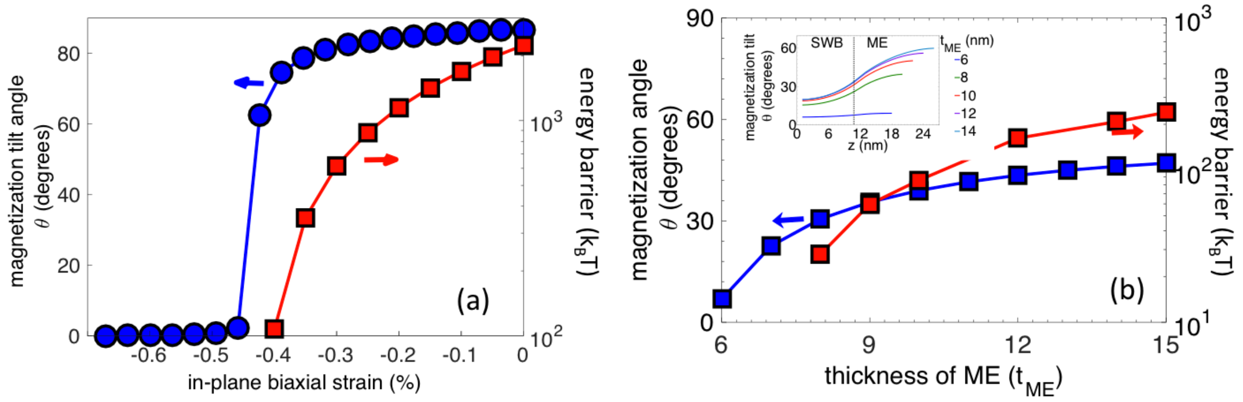

Figure 5: Magnetization tilt angle from the out-of-plane axis and the energy barrier between the stable zero-voltage magnetic states as a function of the (a) in-plane biaxial strain, and (b) thickness of the CoFe layer of the ME cell exchange coupled to the PMA SWB (Inset shows the magnetization profile or the tilt angle through the thickness of the PMA SWB-ME cell exchange-spring structure).

S10. Asymmetric tilted distribution of switching success as a function of detected $< \phi >$

As shown in Fig. 6(d) of the main text, the distribution of the switching success as a function of the detected phase of the spin wave ($<\phi>$) is asymmetric with respect to the y-axis (line joining 90° and 270°). This tilted distribution can be explained by examining the energy landscape and the corresponding constant energy trajectories in the presence of a small built-in strain or exchange-spring system as shown in Fig. 6(a). The constant energy orbits go clockwise around the energy maxima and anti-clockwise around the energy minima [50]. In the presence of damping, the dynamical evolution of magnetization closely follows the orbits, resulting in magnetization switching trajectories as shown in Fig. 6(b). Note that the distribution of the initial angles of the magnet, which will dictate switching to either +x or -x direction, is now not centered around the 0° or 180° (x axis) but around the separatrix which separates the two types of constant energy trajectories (high energy orbits around maxima and low energy orbits around minima). The resultant tilted distribution under zero-thermal noise is shown in Fig. 6(c). Upon adding the thermal noise, the angles in the highlighted zone at the boundary gives rise to non-deterministic switching probability resulting in a lower switching success as shown Fig. 6(d) of the main text.

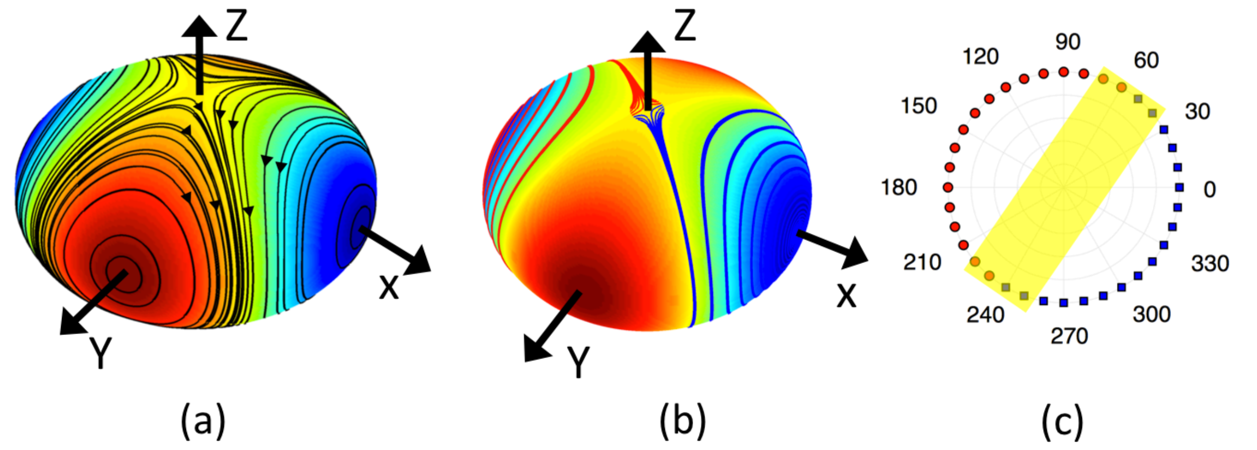

Figure 6: (a) Energy landscape and constant energy trajectories of the nanomagnet in the presence of a small built-in strain or exchange-spring system

S11. Comparison between 1D and full 3D micromagnetic simulation

The full 3D stochastic micromagnetic simulation can be computationally demanding when performing Monte Carlo simulations for thermal reliability. Hence, we resort to a 1D micromagnetic simulation for the case of built-in strain (discretization only along the length) and a 2D simulation for exchange-spring system (discretization along the length and thickness). Such an approximation holds when considering a relatively narrow SWB where the spin waves are uniformly excited along the width of the SWB and propagate only along the length and the higher order width modes are not excited. To further corroborate our assumption, we compare our 1D simulation for built-in strain against the full 3D simulation for a clocking time sweep between 205 ps and 250 ps and show good qualitative agreement between the two in terms of switching success as a function of the clocking time (fig. 7a) and detected phase (fig. 7b). Note that, since we define the magnetization of the ME cell as a spatially averaged quantity, the exactly values of the detected amplitude and phase as a function of time varies a little between the two approaches hence the shift in Fig. 7(a). The result for error-free logic functionality achieved if the detected phase falls within the window from 280° through 0 to 20°, i.e. 100 ^{o}, or from 100° to 200°. still holds when performing 3D simulation (Fig. 7(b)).

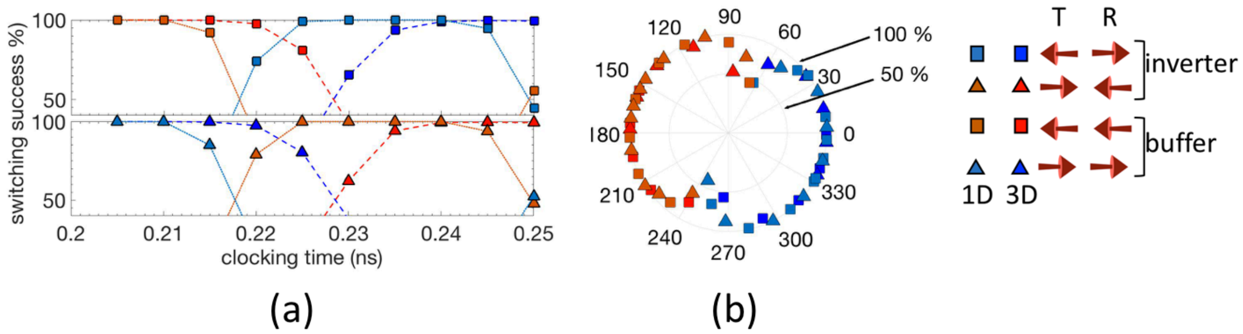

Figure 7. Comparison between 1D and full 3D micromagnetic simulations for switching success as a function of the clocking time (a) and detected phase (b)

S12. Approximate estimation of error rate

Estimation of error rate is essential for ensuring the thermal reliability of a device. As mentioned in the methods of the main text, we 1000 Monte Carlo micromagnetic simulations in OOMMF for each data point to determine the probability of error-free logic functionality. Using such brute force technique to compute error rate below 10^{-3} can become computationally demanding and extreme tails of error-rate cannot be captured in this way. Also, the recently developed "rare-event enhancement" (REE) technique for micromagnetics [51] cannot be trivially applied to our fast picosecond magnetization switching dynamics. Hence, we resort to an equivalent single domain approach of modeling the SW detector [52]. Assuming a distribution of the initial angles θ and ϕ as shown in the insets of Fig. 8 to mimic the effect of arriving spin wave, we let the single domain magnet fall towards an energy minimum. Performing single domain stochastic LLG simulation gave us an error-rate of less than 10^{-5} for up to 10^{5} brute force trials. We further attempted to capture the extreme tails of error-rate by using the REE technique that artificially enhances the rate of occurrence of low-probability events while proportionately reducing their weights to reach an error-rate of less than 10^{-9} as shown in Fig. 8. An alternative approach is to use the Fokker-Planck equation. However, note that still a single domain approximation has to be made while using the Fokker-Planck method. Also note that unlike the case of a PMA magnet, the approximate analytical expression or numerical solution of a 1-D Fokker-Planck cannot be used and one has to resort to a numerical solution of a 2-D Fokker-Planck using FDM or FEM method and is beyond the scope of this work.

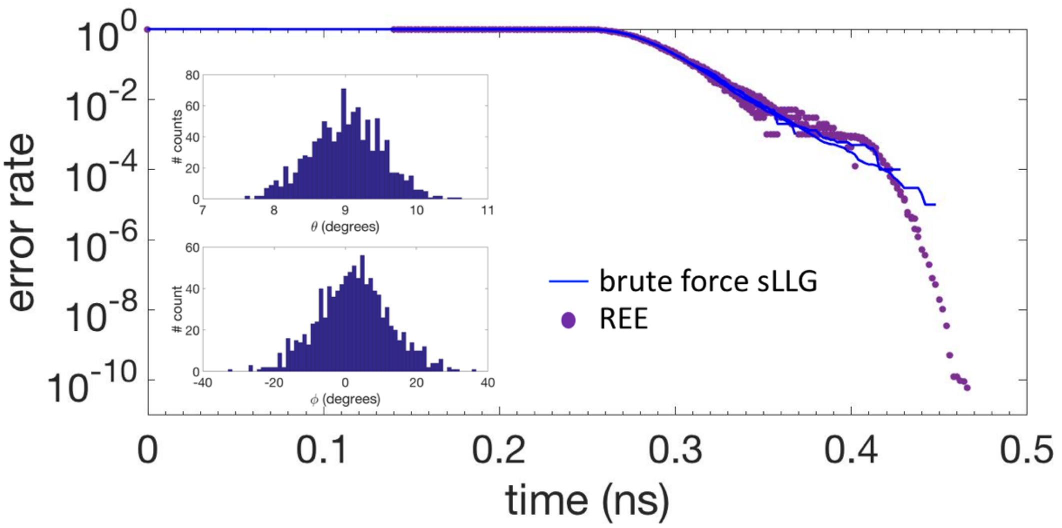

Figure 8. Approximate error rate estimation using equivalent single domain magnetization and performing brute force sLLG simulation and REE technique.

References

1 Dutta, S. *et al.* Non-volatile clocked spin wave interconnect for beyond-CMOS nanomagnet pipelines. *Scientific reports* **5** (2015).

2 Wu, T. *et al.* Electric-poling-induced magnetic anisotropy and electric-field-induced magnetization reorientation in magnetoelectric Ni/(011)[Pb (Mg1/3Nb2/3) O3](1-x)-[PbTiO3] x heterostructure. *Journal of applied physics* **109**, 07D732 (2011).

3 Jungblut, R., Johnson, M., Aan de Stegge, J., Reinders, A. & Den Broeder, F. Orientational and structural dependence of magnetic anisotropy of Cu/Ni/Cu sandwiches: Misfit interface anisotropy. *Journal of Applied Physics* **75**, 6424-6426 (1994).

4 Shiota, Y. *et al.* Induction of coherent magnetization switching in a few atomic layers of FeCo using voltage pulses. *Nature materials* **11**, 39-43 (2012).

5 Ghidini, M. *et al.* Non-volatile electrically-driven repeatable magnetization reversal with no applied magnetic field. *Nature communications* **4**, 1453 (2013).

6 Daalderop, G., Kelly, P. & Den Broeder, F. Prediction and confirmation of perpendicular magnetic anisotropy in Co/Ni multilayers. *Physical review letters* **68**, 682 (1992).

7 Johnson, M., Bloemen, P., Den Broeder, F. & De Vries, J. Magnetic anisotropy in metallic multilayers. *Reports on Progress in Physics* **59**, 1409 (1996).

8 Bloemen, P., De Jonge, W. & Den Broeder, F. Magnetic anisotropies in Co/Ni (111) multilayers. *Journal of applied physics* **72**, 4840-4844 (1992).

9 You, L., Sousa, R., Bandiera, S., Rodmacq, B. & Dieny, B. Co/Ni multilayers with perpendicular anisotropy for spintronic device applications. *Applied Physics Letters* **100**, 172411 (2012).

10 Den Broeder, F., Janssen, E., Hoving, W. & Zeper, W. Perpendicular magnetic anisotropy and coercivity of Co/Ni multilayers. *IEEE transactions on magnetics* **28**, 2760-2765 (1992).

11 Verba, R., Tiberkevich, V., Krivorotov, I. & Slavin, A. Parametric excitation of spin waves by voltage-controlled magnetic anisotropy. *Physical Review Applied* **1**, 044006 (2014).

12 Verba, R., Carpentieri, M., Finocchio, G., Tiberkevich, V. & Slavin, A. Excitation of propagating spin waves in ferromagnetic nanowires by microwave voltage-controlled magnetic anisotropy. *Scientific reports* **6** (2016).

13 Wu, T. *et al.* Electrical control of reversible and permanent magnetization reorientation for magnetoelectric memory devices. *Applied Physics Letters* **98**, 262504 (2011).

14 Wu, T. *et al.* Giant electric-field-induced reversible and permanent magnetization reorientation on magnetoelectric Ni/(011)[Pb (Mg1/3Nb2/3) O3](1-x)-[PbTiO3] x heterostructure. *Applied Physics Letters* **98**, 2504 (2011).

15 Streubel, R., Köhler, D., Schäfer, R. & Eng, L. M. Strain-mediated elastic coupling in magnetoelectric nickel/barium-titanate heterostructures. *Physical Review B* **87**, 054410 (2013).

16 Gepra¨ gs, S., Brandlmaier, A., Opel, M., Gross, R. & Goennenwein, S. Electric field controlled manipulation of the magnetization in Ni/BaTiO3 hybrid structures. *Applied Physics Letters* **96**, 142509 (2010).

17 Zheng, H. *et al.* Multiferroic BaTiO3-CoFe2O4 nanostructures. *Science* **303**, 661-663 (2004).

18 Zheng, H. *et al.* Three-dimensional heteroepitaxy in self-assembled BaTiO3-CoFe2O4 nanostructures. *Applied Physics Letters* **85**, 2035-2037 (2004).
19 Zheng, H., Kreisel, J., Chu, Y.-H., Ramesh, R. & Salamanca-Riba, L. Heteroepitaxially enhanced magnetic anisotropy in BaTiO3–CoFe2O4 nanostructures. *Applied physics letters* **90**, 113113 (2007).
20 Chopdekar, R. & Suzuki, Y. Magnetoelectric coupling in epitaxial CoFe2O4 on BaTiO3. *Applied physics letters* **89**, 2506 (2006).
21 Yang, J. *et al.* Electric field manipulation of magnetization at room temperature in multiferroic CoFe2O4/Pb (Mg1/3Nb2/3) 0.7 Ti0. 3O3 heterostructures. *Applied physics letters* **94**, 212504 (2009).
22 Ding, H., Cheah, J. W., Chen, L., Sritharan, T. & Wang, J. Electric-field control of magnetic properties of CoFe 2 O 4 films on Pb (Mg 1/3 Nb 2/3) O 3–PbTiO 3 substrate. *Thin Solid Films* **522**, 420-424 (2012).
23 Pertsev, N. Giant magnetoelectric effect via strain-induced spin reorientation transitions in ferromagnetic films. *Physical Review B* **78**, 212102 (2008).
24 Hall, R. Single crystal anisotropy and magnetostriction constants of several ferromagnetic materials including alloys of NiFe, SiFe, AlFe, CoNi, and CoFe. *Journal of Applied Physics* **30**, 816-819 (1959).
25 Hall, R. Magnetic anisotropy and magnetostriction of ordered and disordered cobalt-iron alloys. *Journal of Applied Physics* **31**, S157-S158 (1960).
26 Hunter, D. *et al.* Giant magnetostriction in annealed Co1− xFex thin-films. *Nature communications* **2**, 518 (2011).
27 Cavaco, C., Van Kampen, M., Lagae, L. & Borghs, G. A room-temperature electrical field–controlled magnetic memory cell. *Journal of materials research* **22**, 2111-2115 (2007).
28 Zhang, S. *et al.* Giant electrical modulation of magnetization in Co40Fe40B20/Pb (Mg1/3Nb2/3) 0.7 Ti0. 3O3 (011) heterostructure. *Scientific reports* **4**, 3727 (2014).
29 Lei, N. *et al.* Magnetization reversal assisted by the inverse piezoelectric effect in Co-Fe-B/ferroelectric multilayers. *Physical Review B* **84**, 012404 (2011).
30 Chen, Y., Fitchorov, T., Vittoria, C. & Harris, V. Electrically controlled magnetization switching in a multiferroic heterostructure. *Applied Physics Letters* **97**, 052502 (2010).
31 Liu, M. *et al.* Giant electric field tuning of magnetic properties in multiferroic ferrite/ferroelectric heterostructures. *Advanced Functional Materials* **19**, 1826-1831 (2009).
32 Park, J. H., Jeong, Y. K., Ryu, S., Son, J. Y. & Jang, H. M. Electric-field-control of magnetic remanence of NiFe 2 O 4 thin film epitaxially grown on Pb (Mg 1/3 Nb 2/3) O 3–PbTiO 3. *Applied Physics Letters* **96**, 192504 (2010).
33 Brintlinger, T. *et al.* In situ observation of reversible nanomagnetic switching induced by electric fields. *Nano letters* **10**, 1219-1223 (2010).
34 Wang, H. *et al.* Understanding strong magnetostriction in Fe100− xGax alloys. *Scientific reports* **3** (2013).
35 Parkes, D. *et al.* Magnetostrictive thin films for microwave spintronics. *Scientific reports* **3** (2013).

36 Fitchorov, T. *et al.* Tunable fringe magnetic fields induced by converse magnetoelectric coupling in a FeGa/PMN-PT multiferroic heterostructure. *Journal of Applied Physics* **110**, 123916 (2011).
37 Liu, M. *et al.* Electrically induced enormous magnetic anisotropy in Terfenol-D/lead zinc niobate-lead titanate multiferroic heterostructures. *Journal of Applied Physics* **112**, 063917 (2012).
38 Hockel, J. L., Wu, T. & Carman, G. P. Voltage bias influence on the converse magnetoelectric effect of PZT/Terfenol-D/PZT laminates. *Journal of Applied Physics* **109**, 064106 (2011).
39 Roy, K., Bandyopadhyay, S. & Atulasimha, J. Switching dynamics of a magnetostrictive single-domain nanomagnet subjected to stress. *Physical Review B* **83**, 224412 (2011).
40 Park, S. & Shrout, T. R. Ultrahigh strain and piezoelectric behavior in relaxor based ferroelectric single crystals. *Journal of Applied Physics* **82** (1997).
41 Nagarajan, V. Scaling of the piezoelectric response in ferroelectric nanostructures: An effective clamping stress model. *Applied Physics Letters* **87**, 242905 (2005).
42 Hu, J.-M. *et al.* Purely electric-field-driven perpendicular magnetization reversal. *Nano letters* **15**, 616-622 (2015).
43 Chikazumi, S. & Graham, C. D. *Physics of Ferromagnetism 2e*. (Oxford University Press on Demand, 2009).
44 Hsu, C.-J., Hockel, J. L. & Carman, G. P. Magnetoelectric manipulation of domain wall configuration in thin film Ni/[Pb (Mn1/3Nb2/3) O3] 0.68-[PbTiO3] 0.32 (001) heterostructure. *Applied Physics Letters* **100**, 092902 (2012).
45 Maruyama, T. *et al.* Large voltage-induced magnetic anisotropy change in a few atomic layers of iron. *Nature nanotechnology* **4**, 158-161 (2009).
46 Wang, W.-G., Li, M., Hageman, S. & Chien, C. Electric-field-assisted switching in magnetic tunnel junctions. *Nature materials* **11**, 64-68 (2012).
47 Zhu, J. *et al.* Voltage-induced ferromagnetic resonance in magnetic tunnel junctions. *Physical review letters* **108**, 197203 (2012).
48 Alzate, J. G. *et al.* in *Electron Devices Meeting (IEDM), 2012 IEEE International.* 29.25. 21-29.25. 24 (IEEE).
49 Nikonov, D. E. & Young, I. A. Benchmarking spintronic logic devices based on magnetoelectric oxides. *Journal of Materials Research* **29**, 2109-2115 (2014).
50 Mayergoyz, I. D., Bertotti, G. & Serpico, C. *Nonlinear magnetization dynamics in nanosystems*. (Elsevier, 2009).
51 Roy, U., Pramanik, T., Register, L. F. & Banerjee, S. K. Write error rate of spin-transfer-torque random access memory including micromagnetic effects using rare event enhancement. *arXiv preprint arXiv:1603.08512* (2016).
52 Dutta, S., Nikonov, D. E., Manipatruni, S., Young, I. A. & Naeemi, A. Phase-dependent deterministic switching of magnetoelectric spin wave detector in the presence of thermal noise via compensation of demagnetization. *Applied Physics Letters* **107**, 192404 (2015).
